# Prognostic and Clinicopathological Significance of Downregulated E-Cadherin Expression in Patients with Non-Small Cell Lung Cancer (NSCLC): A Meta-Analysis

**DOI:** 10.1371/journal.pone.0099763

**Published:** 2014-06-30

**Authors:** Yan-Long Yang, Ming-Wu Chen, Lei Xian

**Affiliations:** Department of Cardiothoracic Surgery, the First Affiliated Hospital of Guangxi Medical University, Nanning, Guangxi Zhuang Autonomous Region, China; University of Barcelona, Spain

## Abstract

**Background:**

Many studies have investigated the prognostic role of E-cadherin in patients with NSCLC; however, the result still remains inconclusive. An up-to data system review and meta-analysis was necessary to give a comprehensive evaluation of prognostic role of E-cadherin in NSCLC.

**Methods:**

Eligible studies were searched in Pubmed, Embase and Web of Science databases. The inclusion criteria were studies that assessed the relationship between E-cadherin expression detected by immunohistochemistry (IHC) and the prognosis or clinicopathological features in patients with NSCLC. Subgroup analysis according to race, percentage of reduced/negative E-cadherin expression, histological type, and sample size were also conducted. Odds ratio (OR) or hazard ratio (HR) with 95% confidence interval (CI) were calculated to examine the risk or hazard association.

**Results:**

A total of 29 studies including 4010 patients were qualified for analysis. The analysis suggested that downregulated E-cadherin expression was significant associated with unfavorable overall survival (OS) and disease-free survival/progression-free survival (DFS/PFS) in patients with NSCLC. Subgroup analysis by race, percentage of reduced/negative E-cadherin expression, sample size also found the significant association in OS. When only the stage I NSCLC were considered, downregulated E-cadherin expression still had an unfavorable impact on OS. Additionally, downregulated E-cadherin expression was significantly associated with differentiation grade, lymphnode metastasis, vascular invasion, and TNM stage.

**Conclusion:**

Downregulated E-cadherin expression detected by IHC seems to correlate with tumour progression and could serve as an important prognostic factor in patients with NSCLC.

## Introduction

Lung cancer still remains the most common cancer and the most common cause of cancer-related death worldwide. There are 1,820,000 new cases and 1,590,000 death around the world according to the International Agency for Research on Cancer (IARC) in 2012 [1]. About 85% lung cancers were non-small cell lung cancers [2], and approximately two thirds of NSCLC cases were diagnosis at locally advanced (27.6%) or metastatic (38.1%) disease as the typically asymptomatic at early stages [3]. It is well known that pathologic TNM category, age, sex, and cell type are all important prognostic factors for the patients with NSCLC [4]. The advances in molecular biology have enabled researchers to focus on molecular or biological markers in NSCLC.

E-cadherin, a calcium-dependent cell-cell adhesion molecule, is closely linked to the actin cytoskeleton and plays a key role in the maintenance of tissue integrity by the formation of adherens junctions [5]. Loss or dysfunction of E-cadherin is associated with an invasive phenotype in numerous cancers [6]. This evidence indicated that E-cadherin may play an important role in the development and progression of NSCLC and might associate with poor prognosis in patients with NSCLC. Recently, many studies have explored the prognostic role and clinicopathological outcomes in patients with NSCLC, but the results remains controversial. Some studies showed that patients with reduced E-cadherin expression may associated with progression and poor survival; other studies could not confirm this. Due to the limited sample size and static power in individual study, a meta-analysis is necessary to comprehensively evaluate the prognostic and clinicopathological significance of E-cadherin expression in patients with NSCLC.

## Materials and Methods

### Literature search

A comprehensive literature search was conducted in the databases of PubMed, EMBASE and Web of Science. The last search time was Feb 28, 2014. The following terms and combinations were used to identify studies: “E-cadherin”, “CDH1”, “lung cancer”, “lung neoplasm” and “prognosis”. Furthermore, references of retrieved articles and reviews were manually screened for additional studies.

### Inclusion and exclusion criteria

The inclusion criteria were applied to identify the eligible studies: (1) human-based investigations; (2) pathologically confirmed non-small cell lung cancer; (3) articles with full texts published in English; (4) to detect E-cadherin expression in the primary tumor tissues by immunohistochemistry (IHC) assay; (5) to evaluate the correlation between E-cadherin expression and OS, DFS/PFS, or clinicopathological parameters; (6) to provided sufficient information to estimate hazard ratio (HR) or odds ratio (OR) and their 95% confidence intervals (CIs). The exclusion criteria were as the follows: (1) studies published in non-English; (2) cell line and animal studies, case reports, letters, reviews or meta-analysis; (3) studies in which necessary data were not provided; (4) for overlapped studies, the studies with low quality were excluded.

### Data extraction

Two investigators (YL Yang and MW Chen) independently reviewed the eligible studies and extracted the following data: surname of the first author, publication year, country, ethnicity, sample size, disease stage, histology type, assay method, cut off value, distribution of reduced/negative E-cadherin expression and the outcomes. All data were then examined by two investigators independently (YL Yang and MW Chen). Disagreements were resolved by discussion among all authors.

### Quality Assessment

The quality of the methodology of the included studies was assessed by the Newcastle-Ottawa scale (NOS) recommended by the Cochrane Non-Randomized Studies Methods Working Group [7]. Studies with five or more stars were defined as high quality studies. Quality assessment was performed by two investigators (YL Yang and L Xian) independently. Disagreements were resolved by discussion.

### Statistical Analysis

The impact of E-cadherin expression on survival (OS, DFS/PFS) was measured by the combined HRs and their 95% CIs extracted from each eligible study. The HR and its 95% CI in each eligible study was directly extracted from report, or indirectly estimated by methods described by Tierney [8]. The combined HRs was estimated graphically by Forest plots. For the relationship between E-cadherin expression and clinicopathological parameters, odds ratios (ORs) and their 95% CIs were combined to estimate the effective value. The overall HR/OR and its 95% CI overlap 1 was considered statistically significant and indicated a worse effect for the group with reduced/negative E-cadherin expression. Heterogeneity between studies was detected by the Q test and the I^2^ metric (no heterogeneity: I^2^ = 0%–25%; moderate heterogeneity: 25%–50%; large heterogeneity: 50%–75%; and extreme heterogeneity: 75%–100%) [9]. If P≥0.10 in the Q test or I^2^<50%, the fixed-effect model (the Mantel Haenszel method) [10] was used. Otherwise, random effect model [11] analysis was conducted. Subgroup analysis by different analytical methods (race, percentage of reduced E-cadherin, histological type, HR estimate, and sample size) was performed in the analysis of OS. In addition, publication bias was assessed by the method reported by Begg and Egger [12], [13]. All P values were two-tailed and the P value<0.05 was considered statistically significant. Most of the statistical analyses in this study were conducted by the STATA software (version 11.2; StataCorp, College Station, Texas USA).

## Results

### Eligible Studies

**Figure 1:**
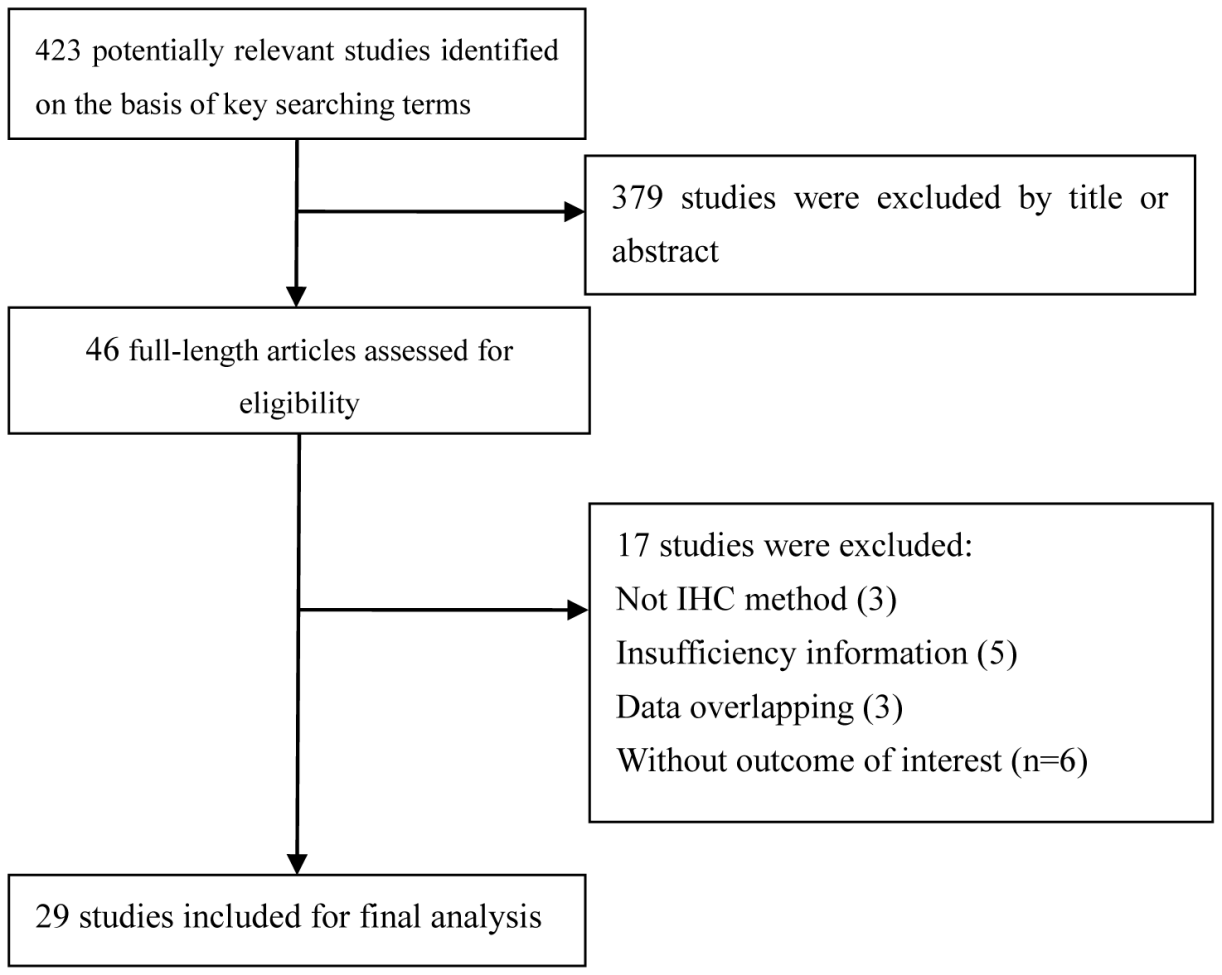
Flow diagram of studies selection procedure.

The present work followed the guidelines for systematic reviews and meta-analyses (PRISMA) (Checklist S1). 423 articles were identified from three databases. After reviewing the titles and abstracts, 379 articles were excluded because they obviously did not meet our selection criteria. The remaining 46 articles were further checked by screening the full texts. 17 studies were excluded for the following reasons: not an IHC method (n = 3), insufficient data (n = 5), without outcome of interest (n = 6), data overlapping (n = 3). Finally, a total of 29 studies [14]–[41] including 4010 patients were qualified for our analysis. The process of article selection is summarized in Figure 1. All 29 studies were assessed by the NOS quality scale and all eligible studies scored highly (with five stars or more). The quality score of the eligible studies can be found in Table S1.

The main characteristics of qualified studies are listed in Table 1. Five studies only investigated the relationship between E-cadherin expression and clinicopathological parameters [37]–[41]. 22 studies investigated the impact of E-cadherin expression on overall survival (OS) [14]–[21], [23], [25]–[29], [31]–[36], and most of them assessed the clinicopathological parameters at the same time [14]–[20], [25], [29], [31], [33], [35]. Among these 22 studies, 17 studies evaluated patients in Asian [14]–[17], [19], [20], [23], [25], [26], [29], [31]–[36], five studies evaluated patients in Caucasian [18], [21], [27], [28], [30]. The percentage of reduced E-cadherin expression was more than 50% in 12 studies [15], [19], [20], [26]–[30], [32], [36], while 10 studies showed percentage of reduced E-cadherin expression was less than 50% [14], [16]–[18], [21], [23], [25], [33]–[35]. 11 studies investigated mostly (>50%) with adenocarcinoma (AD) [15], [17]–[20], [25], [27], [29], [33], [36], eight studies investigated mostly (>50%) with squamous cell carcinoma (SQ) [21], [28]–[32], [34], [35]. HR estimation of 13 studies was given by authors [18], [20], [21], [23], [25]–[27], [30]–[32], [34], while nine were calculated by survival curves [14]–[17], [19], [28], [29], [33], [34], [36]. 13 studies enrolled less than 150 patients [15], [18], [20], [23], [26], [28]–[32], [34], [36] and nine studies included more than 150 patients [14], [16], [17], [19], [21], [25], [27], [33], [35].

**Table 1:** Main characteristics of all studies included in the meta-analysis.

Author,year	Race(country)	No. of patients(M/F)	Stage	Histology distribution	Antibody sorce(Dilution)	Cutoff value	Distribution of reduced E-cadherin (%)	Outcome
Kase,2000 [14]	Asian(Japan)	331(209/122)	I–IV	AD227,SQ104	Transduction(1∶2500)	0.7	42	OS,CP
Kimura,2000 [15]	Asian(Japan)	135(90/45)	I–IV	AD101,SQ25,LCC5,ADS4	DAKO(NR)	0.8	64.4	OS,CP
Lee,2000 [16]	Asian(Taiwan)	207(142/65)	I–III	AD98,SQ74,BAC35	Takara(NR)	0.4	41	OS,CP
Hirata,2001 [17]	Asian(Japan)	249(171/78)	I–IIIA	AD148,SQ90,LCC8	Takara(NR)	0.1	47	OS,CP
Deeb,2004 [18]	Caucasian(USA)	118(63/55)	I–IIIA	AD81,SQ37	Dako(1∶50)	0.1	45	OS,CP
Huang,2005 [19]	Asian(Japan)	173(116/57)	I–III	AD101,SQ58,LCC14	Vector(1∶100)	0.5	57	OS,CP
Tamura,2005 [20]	Asian(Japan)	131(97/34)	I–IIIA	AD81,SQ50	Takara(1∶200)	0.75	54	OS,CP
Al-Saad,2008 [21]	Caucasian(Norway)	335(253/82)	I–IIIA	AD95,SQ191,LCC31,BAC18	Rocklin(NR)	Score = 2	40	OS
Cho,2008 [22]	Asian(Korea)	55(31/24)	I	AD55	Zymed(NR)	0.25	13	DFS
Zhu,2009 [23]	Asian(China)	148(114/34)	IB	AD42,SQ58,LCC2,BAC27,other19	Fuzhou maxim(NR)	ROC	12.8	OS
Ono,2010 [24]	Asian(Japan)	107(71/36)	I	AD74,other33	BD(1∶200)	0.7	17	DFS
Yamashita,2010 [25]	Asian(Japan)	117(77/40)	I	AD78,SQ31,LCC6,other2	BD(1∶200)	0.7	40	OS,CP
Lin,2010 [26]	Asian(China)	185(115/70)	I	SQ63,non-SQ132	Takara(1∶400)	0.5	51	OS
Sterlacci,2010 [27]	Caucasian(Austria)	405(292/113)	I–IV	AD207,SQ126,LCC56,other16	DAKO(NR)	0.25	51	OS
Ucvet,2011 [28]	Caucasian(Turkey)	117(111/6)	I–IV	AD47,SQ62,LCC7,other1	BioGenex(1∶10)	0.5	75	OS
Yu,2011 [29]	Asian(China)	44(22/22)	I–IV	AD44	Santa Cruz(1∶200)	0.75	57	OS,CP
Yu,2011 [29]	Asian(China)	57(42/15)	I–IV	SQ57	Santa Cruz(1∶200)	0.75	60	OS,CP
Richardson,2012 [30]	Caucasian(USA)	38(NR)	I–IV	NR	Danvers(1∶50)	0.1	68	PFS
Wu,2012 [31]	Asian(China)	50(38/12)	I–III	AD14,SQ36	Labvision(NR)	Score = 1	52	OS,CP
Feng,2012 [32]	Asian(China)	103(71/32)	I–IV	AD46,SQ55	Invitrogen(1∶200)	0.1	66	OS
Kim,2013 [33]	Asian(Korea)	193(90/103)	I–III	AD193	BD(1∶200)	Score = 100	18	OS,CP
Zhang X,2013 [34]	Asian(China)	118(76/42)	I–IIIA	AD35,SQ74,other9	BD(1∶400)	0.66	48	OS
Zhang H,2013 [35]	Asian(China)	204(173/31)	I–IIIA	SQ	Invitrogen(1∶200)	Score = 4	49.5	OS,CP
Zhao C,2013 [36]	Asian(China)	119(93/26)	I–IV	AD61,SQ58	Santa Cruz(1∶100)	0.1	53.9	OS
Lim,2000 [37]	Asian(Korea)	175(162/13)	I–IV	AD20.6,SQ79.4	DAKO(NR)	0.5	46.8	CP
Pagaki,2010 [38]	Caucasian(Greece)	70	I–IV	AD23.9,SQ67.1	Menarini(NR)	0.1	55.7	CP
Jin,2012 [39]	Asian(China)	46(28/18)	I–III	AD43.5,SQ37.0	Maixin(1∶50)	0.25	60.9	CP
Shi,2013 [40]	Asian(China)	95(56/39)	I–IV	AD95	Zymed(1∶200)	Score = 4	56.8	CP
Zhao J,2013 [41]	Asian(China)	50(39/11)	I–IV	AD25,SQ19,LCC6	Zymed(NR)	0.6	44	CP

**M**: male; **F**: female; **AD**: adenocarcinoma; **SQ**: squamous cell carcinoma; **OS**: overall survival; **DFS**: disease-free survival; **PFS**: progression-free survival; **CP:** Clinicopathological parameters; **NR**: not reported.

### E-cadherin expression and OS in patients with NSCLC

**Figure 2:**
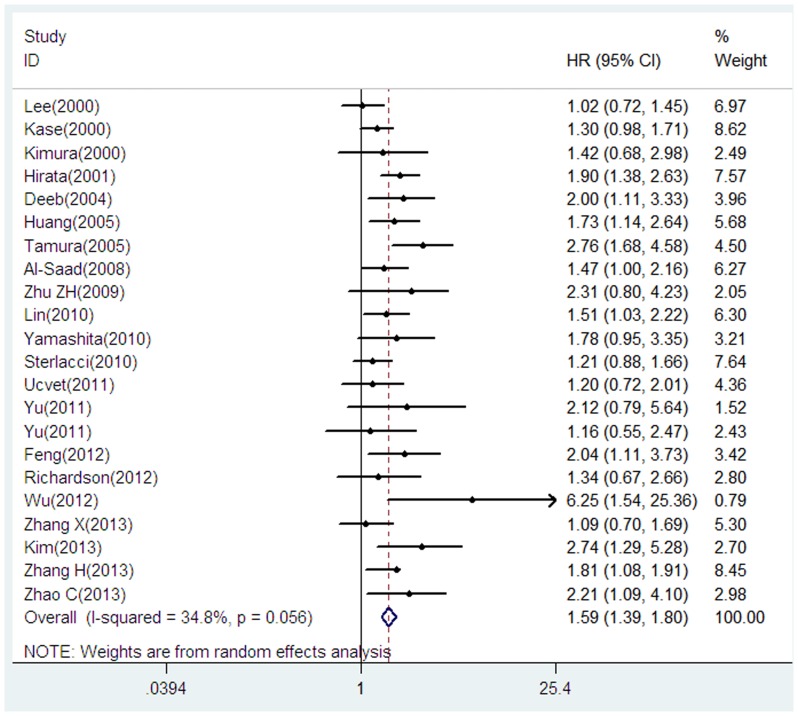
Forrest plot of hazard ratio (HR) for the association of E-cadherin expression with overall survival (OS) in patients with NSCLC.

22 studies including 3575 patients were eligible for the final analysis [14]–[21], [23], [25]–[36]. Our analysis suggested that reduced E-cadherin expression was significantly associated with poor OS when compared to preserved E-cadherin expression (HR = 1.59, 95% CI = 1.39–1.80, *p*<0.001), with moderate heterogeneity between studies (I^2^ = 34.8%, P = 0.056) (Figure 2).

Further subgroup analysis by race suggested that both Asian and Caucasian patients with reduced E-cadherin had a significant impact on OS (Asian: HR = 1.67, 95% CI = 1.42–1.96, *p*<0.001, I^2^ = 41.5%, P = 0.038; Caucasian: HR = 1.37, 95% CI = 1.12–1.66, *p* = 0.002, I^2^ = 0.0%, P = 0.592). When grouped according to the percentage of reduced E-cadherin expression, both studies with reduced E-cadherin >50% and ≤50% suggested the significant results (reduced E-cadherin ≤50%: HR = 1.55, 95% CI = 1.30–1.86, *p*<0.001, I^2^ = 45.7%, P = 0.056; reduced E-cadherin >50%: HR = 1.58, 95% CI = 1.36–1.85, *p*<0.001, I^2^ = 28.9%, P = 0.162). We then focused on the predominant histological type in each study. When we limited the analysis to the studies investigating mostly (>50%) with AD, the pooled HR was 1.65 (95% CI = 1.44–1.89, *p*<0.001, I^2^ = 33.2%, P = 0.133). When we limited the analysis to the studies investigating mostly (>50%) with SQ, the pooled HR was 1.52 (95% CI = 1.29–1.80, *p*<0.001, I^2^ = 29.6%, P = 0.192). When the HRs derived from direct reports from 13 evaluable studies were aggregated, the combined HR was 1.63 (95% CI = 1.43–1.86, *p*<0.001), with moderate heterogeneity between studies (I^2^ = 34.3%, P = 0.108); when the survival data calculated indirectly from Kaplan-Meier based survival curve in nine studies were pooled, the combined HR was 1.45 (95% CI = 1.25–1.67) (*p*<0.001), with moderate heterogeneity between studies (I^2^ = 35.6%, P = 0.133). Subgroup analysis on sample size did not alter the significant prognostic impact of downregulated E-cadherin expression (Table 2).

**Table 2:** Main meta-analysis results of E-cadherin expression in patients with NSCLC.

Analysis	No.of studies(No. of patients)	HR(95%CI)	*P*	Model	Heterogeneity		Publication bias	
					I^2^ (%)	P_het_	Begg's *p*	Egger's *p*
**OS**	22(3574)	1.59(1.39,1.80)	<0.001	R	34.8	0.056	0.159	0.051
Subgroup 1: race								
Asian	17(2561)	1.67(1.42,1.96)	<0.001	R	41.5	0.038	0.266	0.078
Caucasian	5(1013)	1.37(1.12,1.66)	0.002	F	0.0	0.592	0.462	0.483
Subgroup2: percentage of reduced E-cadherin								
≤50%	10(2085)	1.55(1.30,1.86)	<0.001	R	45.7	0.056	0.474	0.301
>50%	12(1489)	1.58(1.36,1.85)	<0.001	F	28.9	0.162	0.373	0.109
Subgroup3: histology type								
AD(>50)		11(2083)	1.65(1.44,1.89)	<0.001	F	33.2	0.133	0.436
SQ(>50)		8(1022)	1.52(1.29,1.80)	<0.001	F	29.6	0.192	0.902
Subgroup4: HR estimate								
Sur. Curve	9(1506)	1.45(1.25,1.67)	<0.001	F	35.6	0.133	0.754	0.507
HR reported	13(2068)	1.63(1.43,1.86)	<0.001	F	34.3	0.108	0.2	0.064
Subgroup5: sample size								
>150	9(2282)	1.52(1.28,1.80)	<0.001	R	45.7	0.065	0.348	0.312
≤150	13(1292)	1.65(1.40,1.95)	<0.001	F	27.4	0.168	0.36	0.136
**OS for stage I patients**	6(717)	1.43(1.14,1.79)	0.002	F	15.5	0.314	0.26	0.281
**DFS/PFS**	4(403)	1.58(1.21,2.05)	0.001	F	20.6	0.286	0.734	0.521
**Clinicopathological parameters**		**OR(95%CI)**						
Histology(AD vs SQ)	12(1371)	0.95(0.65,1.39)	0.795	R	59.6	0.004	0.374	0.264
Differentiation(M/P vs W)	13(1950)	1.71(1.15,2.53)	0.008	R	68.6	<0.001	0.2	0.061
Tumor size(T1/T2 vs T3/T4)	6(1152)	1.12(0.84,1.50)	0.435	F	45.7	0.101	0.707	0.382
Lymphnode status (Yes vs. No)	10(1578)	2.07(1.42,3.02)	0.001	R	71.8	<0.001	0.21	0.099
Pleural invasion(Yes vs No)	3(366)	4.29(0.82,22.44)	0.085	R	85.8	0.001	1	0.901
Vascular invasion(Yes vs No)	2(428)	2.86(1.43,5.73)	0.003	F	0.0	0.755	1	-
Stage (III/IV vs.I/II)	12(1659)	1.87(1.27,2.76)	0.002	R	64.2	0.001	0.086	0.117

**HR**: hazard ratio; **OR**: odds ratio; **95%CI**: 95% confidence interval; **F**: fixed-effect model; **R**: random effect model; **P_het_**: P for Heterogeneity; **AD**: adenocarcinoma; **SQ**: squamous cell carcinoma; **OS**: overall survival; **DFS**: disease-free survival; **PFS**: progression-free survival; **W**: well differentiation; **M**: moderate differentiation; **P**: poor differentiation.

### E-cadherin expression and OS in patients with stage I NSCLC

**Figure 3:**
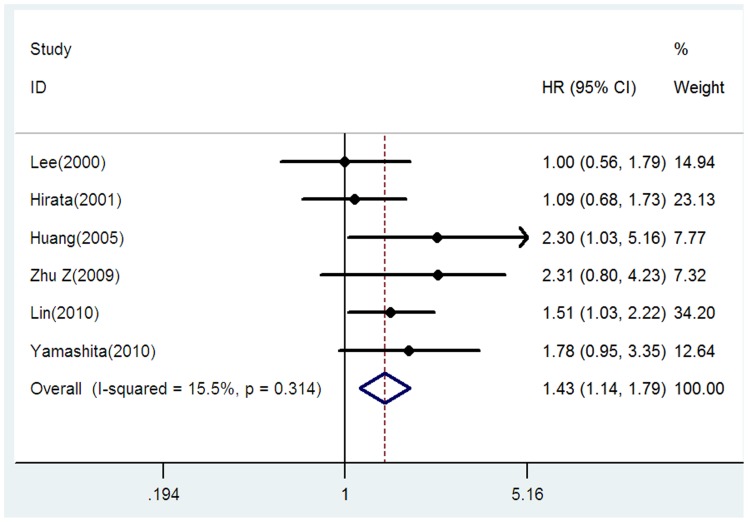
Forrest plot of hazard ratio (HR) for the association of E-cadherin expression with overall survival (OS) in patients with stage I NSCLC.

We separately analyzed the studies with stage I NSCLC. In these six studies with 717 patients [16], [17], [19], [23], [25], [26], the combined HR was 1.43 (95% CI = 1.14–1.79, *p* = 0.002), without heterogeneity between studies (I^2^ = 15.5%, P = 0.314), indicating that reduced E-cadherin expression had significant impact on survival in patients with stage I NSCLC (Figure 3).

### E-cadherin expression and DFS/PFS in patients with NSCLC

**Figure 4:**
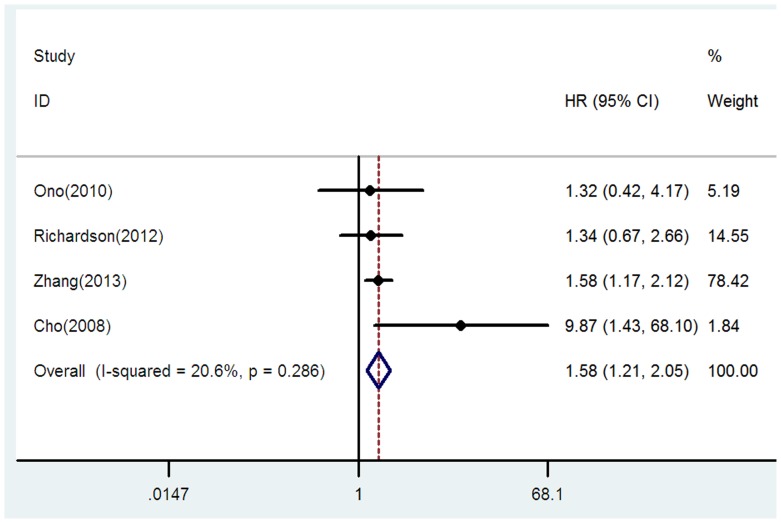
Forrest plot of hazard ratio (HR) for the association of E-cadherin expression with disease-free survival/progression-free survival (DFS/PFS) in patients with NSCLC.

Four studies including 403 patients were eligible for the final analysis [22], [24], [30], [35]. Only one studies evaluated PFS [30] and the remaining studies evaluated DFS. Our analysis suggested that reduced E-cadherin expression was significant associated with worse DFS/PFS when compared to reserved E-cadherin expression (HR = 1.58, 95% CI = 1.21–2.05, *p* = 0.001, I^2^ = 20.6, P = 0.286) (Figure 4).

### E-cadherin expression and clinicopathological parameters in patients with NSCLC

The following clinicopathological parameters extracted from studies were collected for analysis: histological type [16], [17], [19], [20], [29], [31], [33], [35], [38]–[41], grade of differentiation [14]–[20], [25], [31], [35], [37]–[39], tumor size [14], [15], [17], [19], [35], [38], lymph node metastasis [14]–[17], [19], [29], [31], [35], [38], [41], pleural invasion [16], [26], [38], vascular invasion [14], [16], and TNM stages [16], [17], [19], [20], [29], [31], [33], [35], [38]–[41]. As showed in Table 2, our analysis suggested that downregulation of E-cadherin was significantly associated with grade of differentiation (moderate/poor vs: well: OR = 1.71, 95% CI = 1.15–2.53, *p* = 0.008, I^2^ = 68.6%, P<0.001), lymphnode metastasis (yes vs no: OR = 2.07, 95% CI = 1.42–3.02, *p* = 0.001, I^2^ = 71.8%, P<0.001), vascular invasion (yes vs no: OR = 2.86, 95% CI = 1.43–5.73, *p* = 0.003, I^2^ = 0.0%, P = 0.755), and TNM stages (III/IV vs. I/II: OR = 1.87, 95% CI = 1.27–2.76, *p* = 0.002, I^2^ = 64.2%, P = 0.001). However, no significant association between downregulation of E-cadherin and histological type, tumor size, and pleural invasion was found (Table 2).

### Publication bias

**Figure 5:**
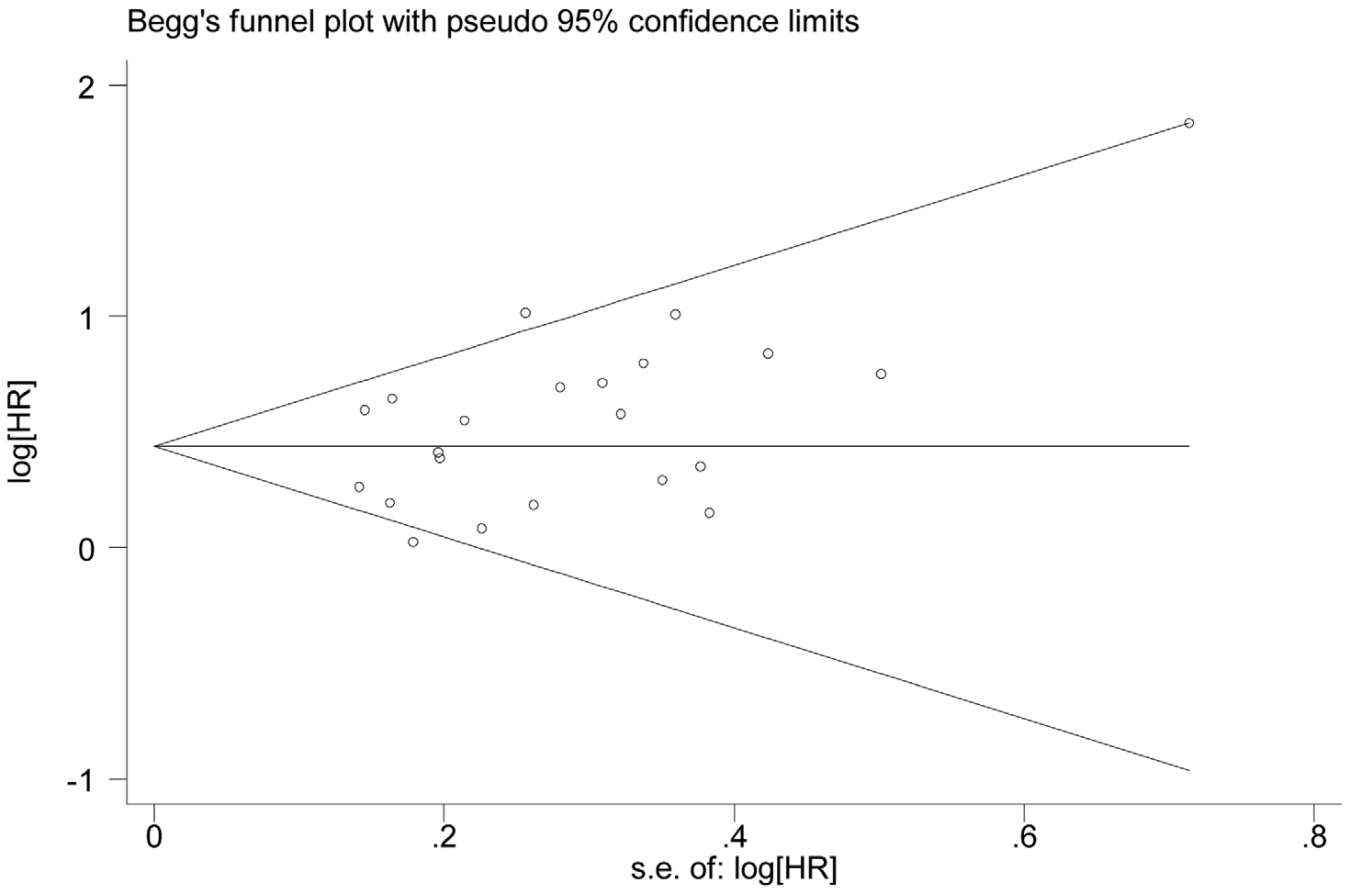
The funnel plot of the meta-analysis of the impact of E-cadherin expression on overall survival (OS) in patients with NSCLC.

Begg's funnel plot and Egger's test were performed to assess the publication bias of studies. As showed in Table 2, no publication bias was detected in all comparisons. The shape of the funnel plot was symmetrical for the all comparisons, Figure 5 showed the funnel plot in the comparison of OS in patients with NSCLC.

## Discussion

In this meta-analysis, we explored the prognostic role of E-cadherin expression in patients with NSCLC. Our analysis suggested that downregulation of E-cadherin was associated with poor OS and DFS/PFS in patients with NSCLC. When stage I NSCLC was separately analyzed, the prognostic impact on OS of downregulated E-cadherin was still significant. In addition, significant correlation was observed between E-cadherin expression and clinicopathological features including grade of differentiation, lymph node metastasis, vascular invasion, and TNM stages.

What are the possible mechanisms of E-cadherin in tumor progression? First, the loss of E-cadherin disrupts adhesion junctions between neighbouring cells and as a result, malignant cells detach from the epithelial-cell layer [42]. Second, loss of E-cadherin has direct effects on signalling pathways involved in tumour-cell migration and tumour growth, including the canonical Wnt signalling pathway and Rho family GTPase-mediated modulation of the actin cytoskeleton [43], [44]. In addition, loss of E-cadherin expression is an epithelial-mesenchymal transition (EMT) hallmark [45], which participates in the progression and metastases of many epithelial tumors [46]. the loss of E-cadherin is frequently correlated with the gain of expression of mesenchymal cadherins, such as N-cadherin, which enhance tumour-cell motility and migration [47]. Hence, the loss of E-cadherin may play a critical role in tumour invasion and metastatic dissemination, not only by changing the adhesive repertoire of a tumour cell, but also by modulating various signalling pathways and transcriptional responses [42].

A previous meta-analysis by Wu et al. [48] had been performed to examine the prognostic role in patients with NSCLC, and the result was consisted with us. However, our study showed the following advancements when compared with previous work. Firstly, our study included larger sample size than previous one. Wu closed their search time on 2011; however, after their work published, additional eight studies including 926 patients were published [29]–[36]. These additional eight studies were included in our analysis, in some degree, our result was more robust and reliable than previous work. Secondly, our study showed lower heterogeneity than Wu's study in the investigation of the impact of E-cadherin expression on OS (34.8% vs 64%). Maybe more studies with larger sample size reduced the heterogeneity. Thirdly, In Wu's study, they did not found the significant association between E-cadherin and OS in stage I NSCLC patients; however, we found this significant association. They may ignore one study by Zhu et al. that they used receiver operating characteristic (ROC) curve analysis to determine the cutoff cutoff score of E-cadherin [23]. Beside, various subgroup analyses were done in our analysis, while in previous study, they only conducted subgroup analysis by race. At last, our study provided more information and gave a comprehensive insight on the role of E-cadherin in the progression of NSCLC. In Wu's study, they only investigated the relationship between E-cadherin expression and OS. However, in our study, we provided the information not only OS, but also DFS/PFS and clinicopathological features. The present study indicated downregulation of E-cadherin was significantly correlated with poor OS and DFS/PFS, additionally, downregulation of E-cadherin was associated with invasive phenotype (including grade of differentiation, lymph node metastasis, vascular invasion, and TNM stages) in NSCLC. Based on the above points, we thought our up-to date meta-analysis was worthwhile and comprehensive.

Various subgroup analyses were done. When we limited to the race, HR estimate, sample size, percentage of reduced/negative E-cadherin, and histological type, all these subgroups suggested the significant association between E-cadherin expression and poor OS. In addition, when we focus to stage I NSCLC, downregulated E-cadherin expression was associated with survival, suggesting this prognostic factor could also be of importance in early-stage NSCLC. What's more, E-cadherin expression was also related to poor DFS/PFS. In additional, downregulated E-cadherin expression was correlated with poor grade of differentiation, positive lymph node metastasis, positive vascular invasion, and advanced TNM stages, indicating downregulated E-cadherin in NSCLC presented invasive phenotypes. As a result, poor survival is very likely the consequence. All these evidence we observed demonstrated that E-cadherin was closely related to progression of NSCLC.

Our analysis provided the evidence that E-cadherin maybe a prognostic factor in NSCLC patients. However, as neoplastic progression is a complex and multiple-step process, E-cadherin may only play a small role. Combining E-cadherin with other biomarkers would be more meaningful and efficient, Also, gene-gene and gene-environment interaction showed be taken into consideration. Beside, E-cadherin may serve as a novel target and the application of individualized management in NSCLC patients. As loss of E-cadherin expression may associate with neoplastic progression, reconstitution of E-cadherin expression maybe an apparent attractive approach for treatment of NSCLC. This would be possible to prevent E-cadherin promoter methylation in some cases [5]. In addition, the signalling pathways such as such as HER receptors (HER2/neu and EGFR) and Notch downstream targets are aberrantly activated in consequence of E-cadherin loss. Since EGFR and Notch inhibitors are already developed as therapeutic agents in diverse tumour models, these targets and associated pathways will create the basis for the development of new therapeutic control in E-cadherin-mediated cancer [5].

Some limitations should be acknowledged.

Firstly, in the studies we included, IHC techniques used to detect protein expression were not the same (including antibody type and concentration, the cutoff value definition). These differences could contribute to the heterogeneity.

Secondly, postoperative adjuvant therapy should be taken into consideration. The included studies showed the different management. Some only received surgery, while others received additional adjuvant chemotherapy and (or) adjuvant radiotherapy. This may be one of the major resources of heterogeneity.

Moreover, the HRs and their 95% CI we extracted from the OS data were not consistent. We have to estimate the HRs by reading the Kaplan-Meier curves because some studies did not report the HRs. Some studies reported the unadjusted HRs while the others provided the adjusted HRs. Moreover, the cofounders they adjusted were not the same for the adjusted HRs. All of these factors more or less contributed to the heterogeneity.

At last, potential publication biases may exist. Articles were not written in English and studies failed to get published because of negative or null results cannot be identified in our literature search and thus were not included in this analysis. In addition, some reports did not provide sufficient data were also excluded from our analysis.

In conclusion, our study indicated that downregulated E-cadherin expression correlate with tumour progression and prognosis of NSCLC patients. E-cadherin might be a predicative factor of progression, and prognosis of patients with NSCLC. With the limitations, heterogeneities, and bias of meta-analysis, our conclusions in this study need to be interpreted with caution. Future large prospective studies with rigorously designed methodology are warranted to confirm our results.

## Supporting Information

Figure S1 PRISMA Flow Diagram.
(DOC)

Table S1 Quality assessment of eligible studies with Newcastle-Ottawa Scale.
(DOCX)

Checklist S1 PRISMA Checklist.
(DOC)
