# Supplementary material for: Prognostic and Clinicopathological Significance of Downregulated E-Cadherin Expression in Patients with Non-Small Cell Lung Cancer (NSCLC): A Meta-Analysis
Source: PLoS One. 2014 Jun 30;9(6):e99763. doi: 10.1371/journal.pone.0099763 (PMC4076188; doi:10.1371/journal.pone.0099763)
Supplement: Figure S1 — PRISMA Flow Diagram. (DOC) [file pone.0099763.s001.doc]

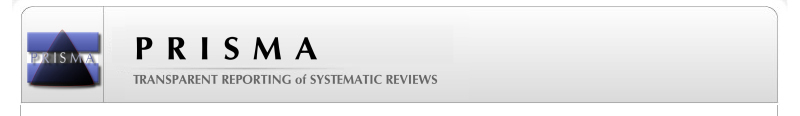
**PRISMA 2009 Flow Diagram**

**Screening**

**Included**

**Eligibility**

**Identification**

Records identified through database searching
(n =423 )

Additional records identified through other sources
(n =0 )

Records after duplicates removed
(n = 423 )

Records screened
(n = 423 )

Records excluded
(n = 379 )

Full-text articles assessed for eligibility
(n = 46 )

Full-text articles excluded, with reasons
(n = 17 )

Studies included in qualitative synthesis
(n =29 )

Studies included in quantitative synthesis (meta-analysis)
(n = 29 )
