# Supplementary material for: Prognostic and Clinicopathological Significance of Downregulated E-Cadherin Expression in Patients with Non-Small Cell Lung Cancer (NSCLC): A Meta-Analysis
Source: PLoS One. 2014 Jun 30;9(6):e99763. doi: 10.1371/journal.pone.0099763 (PMC4076188; doi:10.1371/journal.pone.0099763)
Supplement: Table S1 — Quality assessment of eligible studies with Newcastle-Ottawa Scale. (DOCX) [file pone.0099763.s002.docx]

Table S1.Quality assessment of eligible studies with Newcastle-Ottawa Scale

| Author | Year | Selection | Comparability | Outcome |
| --- | --- | --- | --- | --- |
| Kase | 2000 | ★★★★ | ★★ | ★★ |
| Kimura | 2000 | ★★★ | ★★ | ★★ |
| Lee | 2000 | ★★★ | ★★ | ★★ |
| Hirata | 2001 | ★★★★ | ★★ | ★★ |
| Deeb | 2004 | ★★★ | ★★ | ★★★ |
| Huang | 2005 | ★★★ | ★★ | ★★ |
| Tamura | 2005 | ★★★ | ★ | ★★★ |
| AI-Saad | 2008 | ★★★ | ★★ | ★★★ |
| Cho | 2008 | ★★ | ★ | ★★ |
| Zhu | 2009 | ★★★★ | ★ | ★★★ |
| Ono | 2010 | ★★ | ★ | ★★ |
| Yamashita | 2010 | ★★★ | ★★ | ★★★ |
| Lin | 2010 | ★★★★ | ★★ | ★★★ |
| Sterlacci | 2010 | ★★★★ | ★★ | ★★★ |
| Ucvet | 2011 | ★★★ | ★ | ★★ |
| Yu | 2011 | ★★ | ★★ | ★★ |
| Richardson | 2012 | ★★★ | ★ | ★★ |
| Wu | 2012 | ★★ | ★ | ★★ |
| Feng | 2012 | ★★★ | ★ | ★★ |
| Kim | 2013 | ★★★ | ★★ | ★★ |
| Zhang X | 2013 | ★★ | ★★ | ★★★ |
| Zhang H | 2013 | ★★★★ | ★★ | ★★★ |
| Zhao C | 2013 | ★★ | ★★ | ★★ |
| Lim | 2000 | ★★ | ★ | ★★ |
| Pagaki | 2010 | ★★★ | ★ | ★★ |
| Jin | 2012 | ★★★ | ★★ | ★★ |
| Shi | 2013 | ★★★ | ★★ | ★ |
| Zhao J | 2013 | ★★★ | ★ | ★ |
